# Absence of lunar phobia in European swarming vespertilionid bats

**DOI:** 10.1038/s41598-024-53281-z

**Published:** 2024-02-01

**Authors:** Grzegorz Apoznański, Felix Tuff, Andrew Carr, Alek Rachwald, Ewa Marszałek, Tomasz Marszałek, Justyna Błesznowska, Tomasz Kokurewicz

**Affiliations:** 1 Department of Vertebrate Ecology and Paleontology, Institute of Environmental Biology, Wrocław University of Environmental and Life Sciences, Wrocław, Poland; 2 Tuff Ecology, Petersfield, UK; 3 Forest Ecology Department, Forest Research Institute, Sękocin Stary, Raszyn, Poland

**Keywords:** Ecology, Evolution, Zoology, Environmental sciences

## Abstract

“Lunar phobia” in bats has been widely discussed since its description in tropical bats in 1978. The phenomenon has been frequently contested and supported and was first reported in European bats in 2020. Our study seeks to clarify the debate by describing the relationship between the activity of selected swarming vespertilionid bats (Family: Vespertilionidae) and moonlight levels. To verify a potential connection to the latter, a swarming dataset was analysed in respect of estimated moonlight illumination. Moonlight estimates were based on geographical location and several lunar parameters, to accurately characterise the non-linear relationship between moon phase and illumination (lux). The swarming data consisted of 32 netting and 14 echolocation recording sessions collected between August and October 2014 and 2015. Our data included 3,265 netted bats from 13 species and 15,919 bat calls from 10 confirmed species. Data was collected at the large Central European hibernation/swarming site – Natura 2000 PLH080003 “Nietoperek” in western Poland (N 52.394400, E 15.480600). Generalised linear mixed models (GLMMs) determined insignificant relationships between bats and moonlight illumination. Our analysis confirms an absence of impact of moonlight intensity on swarming bats and thereby rejects the lunar phobia phenomena in at least six insectivorous bat species (*Myotis myotis, M. daubentonii, M. nattereri, M. bechsteinii, Barbastella barbastellus, Plecotus auritus*) swarming in the autumn.

## Introduction

Bats (order Chiroptera) serve as an intuitive example of a nocturnal animal. In fact, most mammals are more active at night, utilising the cover of darkness to avoid predation^1,2^. Represented by over 1400 species, bats constitute the second most diverse mammalian order globally^3^. With such high representation in all but polar ecosystems they are inevitably subjected to predation pressures^4–6^. While individual, small, and manoeuvrable insectivorous bats make rather poor targets for predators, the same species can occur in large numbers during swarming^7–9^, when bats aggregate prior to hibernation for mating and information exchange. Due to the increased density of potential prey and the relatively narrow time window, corresponding to the evening activity, makes swarming bats a valuable seasonal food resource^10,11^. Furthermore, bats can occur in increased numbers^12–15^ when entering and exiting their roosts which typically coincides with dawn and dusk, respectively, when day illumination is still sufficient for diurnal birds of prey to hunt^16,17^. Moreover, owls, as nocturnal predators constitute additional predation threats throughout the night and this risk increases during swarming^16,18,19^. Faced with considerable predation pressure, it is logical to assume that bats have developed countermeasures. One possible avoidance behaviour debated by researchers is “lunar phobia” (negative correlation between activity and moon illumination) to reduce visual detection by predators. Initially documented in Jamaican fruit bats (*Artibeus jamaicensis*)^20^, this relationship has been included in wide range of field guides and research projects and is often accepted during current observation protocols in Europe and around the globe.

https://batmanagement.com/blogs/bat-exclusion-control/bat-house-test-1; https://www2.gov.bc.ca/assets/gov/environment/natural-resource-stewardship/nr-laws-policy/risc/bats.pdf; https://www.rbkc.gov.uk/pdf/Bat_survey_complete_2010.pdf.

Swarming in bats is the phenomenon of collective flights during certain periods of their seasonal activity, during which they demonstrate a high level of social activity. This general term is used to describe, among others, the group flights of some bat species in spring after leaving their hibernacula^21–23^, as well as periodic collective flights around summer colonies^24,25^. However, most often the term refers to autumn swarming, which is considered important for the biology of many species of bats in the temperate zone. Autumn swarming, a not yet fully understood social phenomena first reported in North America^7,8,26^, occurs in late summer and early autumn and consists of temporary aggregations of bats at subterranean hibernation sites. From early autumn bats migrate from summer colonies to hibernacula. At this time various bat species in vicinity of the entrance to the hibernaculum, and inside it, perform mating flights, which are described mainly as circling and racing^27^, often with accompanying vocalization^28,29^. The majority of European bat species exhibit swarming^30–32^, whereby they migrate from summer colonies to potential wintering places where they perform mating flights^27^, often accompanied by vocalisation^28,29^. During this period dense groups of individuals are easy prey for opportunistic predators making this an important period to study anti-predation behaviour in bats, and their responses to environmental changes such as lunar illumination.

Lunar phobia, however well established in tropical frugivorous and sanguivorous bats^20,33,34^, is disputed in research of higher latitude species^17,35–37^. An apparent lack of lunar phobia in “northern” bat species is likely due to several factors including highly mobile foraging strategies, reduced predators relative to the tropics, and extended twilight periods^17^. Despite a growing body of support for an absence of lunar phobia in insectivorous bat species from temperate regions^17,38^ a relatively recent study on European bats found moon illumination negatively influenced the foraging activity of some bat species^39^. The assumption of lunar phobia is still frequently purported e.g. in bat survey guides, where it is sometimes encountered and the discussion remains open, thereby warranting further detailed investigation.

We analysed a dataset comprised of netting and recording results from a swarming study carried out in western Poland during autumn swarming in 2014 and 2015, against moonlight illumination calculations. Frequently moon phase or moon percentage has been used in studies exploring lunar phobia^40^. However, to better quantify ecologically relevant levels of moonlight and the exponential relationship between light intensity and moon phase, we used quantitative moonlight illumination estimates^41^. Our goal was to test the hypothesis that moonlight illumination has an impact on total bat or individual species activity in a period important for bat biology with greater predation pressure, such as autumn swarming.

## Materials and methods

### Study site

The “Nietoperek” Natura 2000 site PLH080003 (https://natura2000.eea.europa.eu/?sitecode=PLH080003&views=Sites_View) is a dedicated bat reserve located in western Poland in Lubuskie voivodeship (central point: E 15.480600, N 52.394400). The protected area covers 7377.37 ha, half of which (46.19%, ca. 3400 ha) is composed of managed coniferous forest, with isolated patches of alder, *Alnus sp*., and ash, *Fraxinus sp.,* growing in depressions along river banks and marshes making them impractical for felling. The remaining half (53.81%) of the area is best described as typical central European agrocenosis composed of fields and shrubland. The most important part of this site for bats is a 32 km long underground network of tunnels and various above ground fortifications constructed in the 30 s by the German Reich as a part of a larger defensive front “Ostwall” or “Festungsfront im Oder-Warthe-Bogen”^42^. In the aftermath of the second world war, at the Yalta Conference, on Joseph Stalin’s demand Polish borders have been redrawn granting territorial gains in the west at the expense of Germany while ceding territory to Soviet Union in the East. Due to that, the German county of Landkreis Meseritz, including forementioned underground system was incorporated into the Republic of Poland. Fortifications, deemed impractical for modern military use, currently serve as a tourist attraction and an annual winter home for up to 40,000 individual bats of 12 species, securing their place as one of the biggest European hibernation sites^42,43^.

### Netting

**Figure 1:**
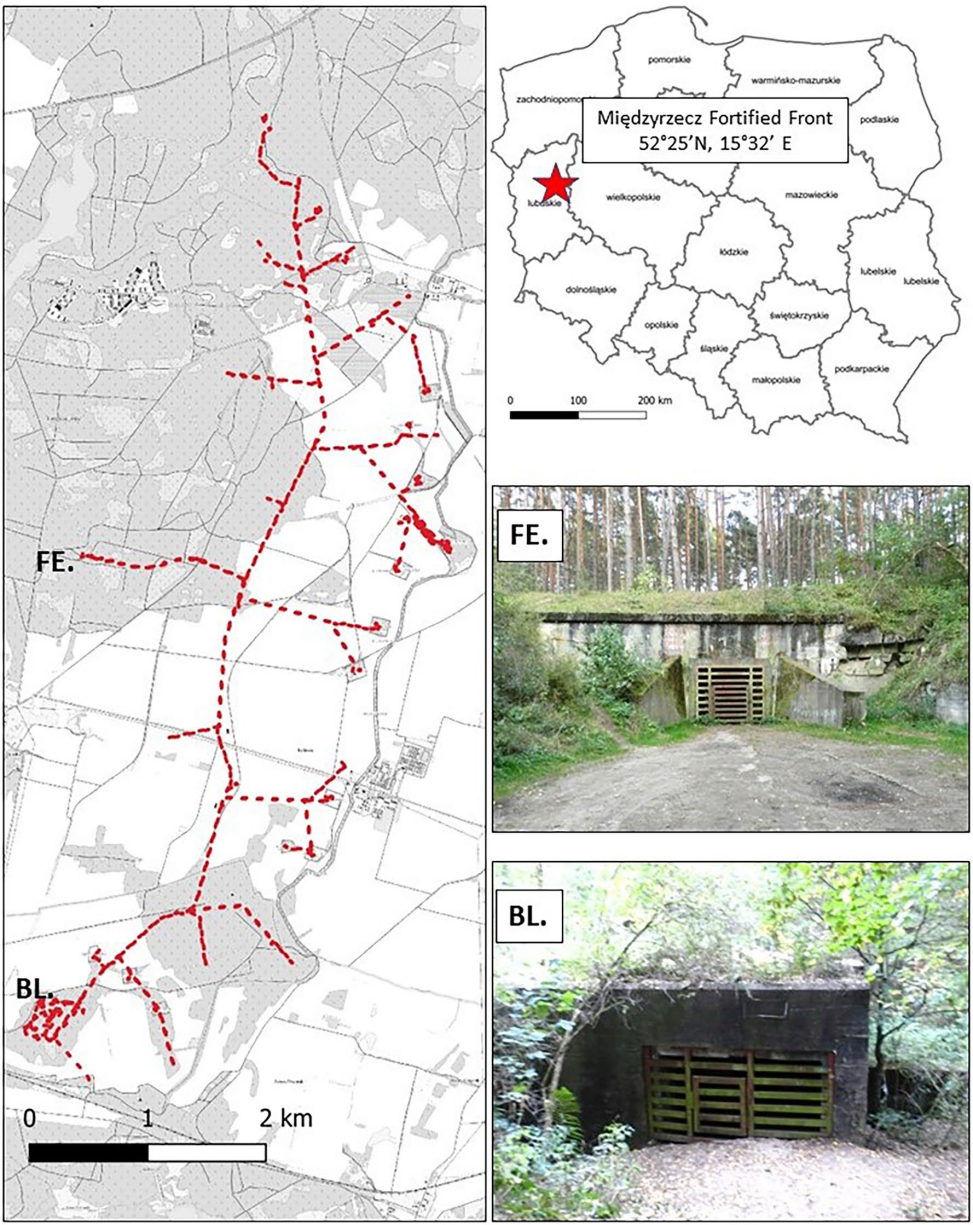
Location of study plots *FE* “Forest Entrance” and *BL* “Boryszyńska Loop” overlayed on the underground system (left), pictures of the plots (right) and their location within Poland (top right).

Netting points were established near two entrance points to the underground network, four kilometres apart—object A64 referred to as Forest Entrance (FE) and an above ground Bunker Pz.T. 2 on Boryszyńska Loop (BL) (Fig. 1). The FE site has a large square opening (ca. 2 × 2 m) leading to the tunnels. Historically, this served as a main entrance for supplies, hence the area in front of it has been clearcut to clear access. Hence, there is full visibility of the open sky at the swarming site. The BL netting point was selected due to its vicinity to a bunker on top of the southern part of the underground system, near the entrance point. The BL site is used in the summer by a big greater mouse-eared bat, *Myotis myotis,* maternity colony exceeding 1000 individuals^42^. Both sites are well established swarming areas. To mitigate illegal tourism both entrance points are gated with a special grill allowing the free movement of bats.

Netting was conducted over four separate sessions, each composed of four netting nights, two per study plot, altering sites each night. Data were collected between August and October 2014 and repeated in 2015 to cover gaps in the season, thereby allowing coverage for the entire swarming period. This resulted in a total of 16 survey nights per site over two years. Netting was conducted in favourable weather conditions (x̄T > 6 °C, wind speed < 4 on the Beaufort scale, with no precipitation, no full cloud cover and no fog). Exact dates and results are presented in Supplementary Materials 1 and 2.

Each night, nets were set up around sunset and dismantled usually around 3 a.m. once bat activity clearly decreased (less than one captured bat per 15 min). The aim of the study was to monitor swarming behaviour, which according to literature is most prominent between 10 p.m. to 2 a.m.^44,45^. Therefore, netting till dawn was deemed unnecessary. The nets themselves were polyester (Avinet TB Mist Net, The United Kingdom), 6 m, 9 m, and 18 m long; with 19 × 19 mm mesh size (double braid). The nets were spread on dedicated three-meter poles. At each location, the network layout was adjusted differently in order to secure optimal area coverage. At the FE, 2 × 9 m nets were erected in a funnel design, perpendicular to the tunnel opening. An additional 18 m net was installed on the entrance roof. At the BL, 1 × 6 m and 1 × 9 m nets were erected adjacent to the entrance. All net locations were considered to permit bats entering and exiting the system whilst targeting swarming bats.

Our study follow ARRIVE guidelines. All experimental protocols were approved by a named institutional licensing committee and were carried out in accordance with relevant guidelines and regulations. In case of our research capture and handling of bats was carried out under license from The Regional Directorate for Environmental Protection (RDOŚ) in Gorzów Wielkopolski WPN-I-6205.34.2014.AI issued on the 07.07.2014 and WPN-I.6401.369.2015.JK issued on the 30.12.2015.

### Bioacoustics

Netting was supplemented with full spectrum recordings using a Pettersson D500x bat detector (Pettersson Elektronik AB, Sweden). A detector was deployed at each netting site for two nights per session. In order to avoid interfering with recording data, recordings were carried out on plots unnetted on those nights. Similarly to netting, a detector was deployed near the entrance point and set to record from sunset until a significant drop in bat activity occurred (usually after 3:00), Detectors were set to automatic triggering (medium/2) with high-pass filter set to 15-kHz, 300-kHz sampling rate and 3-s recording time. Species were manually identified using BatSound (Pettersson Elektronik AB, Sweden) and bioacoustics keys^46^. We defined a bat pass as a single species echolocation sequence no longer than 5 s, with a minimum number of signals constituting two pulses.

### Temperature

Temperature data, consisting of hourly measurements, were obtained from the nearest weather station of the National Institute of Meteorology and Water Management at Lubinicko-Świebodzin, 10 km from our study site. Average temperature from survey start to finish of a netting/recording night was calculated.

### Moonlight estimation

To estimate ecologically relevant levels of relative moonlight illumination, rather than relying on entirely moon phase as a proxy, a model developed by Śmielak was applied^41^. The model accounts for dynamic astronomical parameters, comprising lunar disk brightness*,* moon visibility, atmospheric extinction of light, distance to the moon, and the angle of sunlight reaching the moon. To determine these parameters, site location (E 15.480600, N 52.394400), date, time zone (Warsaw/GMT + 2), sampling interval (15 min), and atmospheric extinction coefficient (0.27) were inputted to the calculatemoonlightstatistics function derived from the moonlit package in R. The extinction coefficient was approximated from the altitude, which was *c.* 100 m above sea level at the netting sites. Using these data, the moonlit package produced values for mean moonlight intensity (lux) per night. This approach accurately estimates moon brightness, however illumination restrictions such as cloud and vegetation cover were not incorporated into the model.

### Statistical analysis

To determine whether night lux levels influenced bat activity, we performed a series of Generalised Linear Mixed Models (GLMMs). Models were completed to assess the influence of moonlight on total netted bat activity, total acoustic bat activity and the six most frequently netted species with sufficient sample sizes. In decreasing order of abundance, these comprised: greater mouse eared (*Myotis myotis*), Daubenton’s bat (*M. daubentonii),* Natterer’s bat (*M. nattereri*), Bechstein’s bat (*M. bechsteinii*), western barbastelle (*Barbastella barbastellus*) and brown long eared bat (*Plecotus auritus*). All bycatch of non-swarming species in nets and on static detectors were removed from the data prior to analysis. A preliminary assessment of the data distribution for each species was made by evaluating histograms.

We first built a set of models that included the number of bat captures (species combined and separated) as response variables and mean moonlight lux levels and site as fixed effects, using the ‘lme4’ R package. For *M. nattereri*, the glmmTMB package was used to account for zero inflated data caused by an excess of nights with no *M. nattereri* captures. Temperature was included as a random effect for all models. We then built a similar set of models that included the number of bats passes recorded by static detectors as the response variable. This was conducted for total bats only due to the reduced reliability in confirming bat species from echolocation calls, particular with bats of the *Myotis* genus. Data were not transformed prior to model building. Resulting models followed the same formula (GLMM = activity ~ lux + site + (1|temperature) with specifications made to error distribution families following model diagnostics. All models used log link functions.

Each model was analysed using the DHARMa R package. Diagnostic testing included checking the distribution of residuals via Q-Q plots and assessing model fit via Kolmogorov–Smirnov tests. The simulation functions in the DHARMa R package were applied to test for dispersion and influential outliers. The results were used to identify the most appropriate error distributions applied to each model (Table 1). The GLMM summaries provided the estimate, standard error (SE), and t/z- values whilst likelihood ratio tests were used to compute *p-*values.

**Table 1:** GLMM outputs of total bats and individual species vs mean moonlight illumination.

Survey method	Response variable	Error distribution	Predictor variable	Estimate	SE	z/t-value	*p*
Netting	Total number of bats	P	Lux	− 0.13	0.88	− 0.15	0.88
	*M. myotis*	NB		− 2.20	0.75	− 2.93	0.34
	*M. daubentonii*	P		1.67	1.12	1.49	0.14
	*M. nattereri*	NB, ZI		− 1.14	4.03	− 0.28	0.78
	*M. bechsteinii*	P		3.46	2.00	1.73	0.10
	*B. barbastellus*	P		1.21	1.55	0.78	0.44
	*P. auritus*	NB		− 6.35	3.70	− 1.72	0.08
Acoustics	Total number of bats	GP		− 0.31	1.67	− 0.19	0.85

*NB* negative binomial, *P* Poisson, *GP* Generalised Poisson, *ZI* zero inflated.

All statistical analyses were performed in R 4.1.2 and RStudio 1.0.143^47^.

## Results

After excluding records of non-swarming species and those with insufficient sample sizes, our analysis was based on a dataset composed of a total of 3206 captures and 15,426 recordings (see Supplementary Materials 1 and 2). Among the captured species, the most numerous was the greater mouse eared bat (31%) followed by Daubenton’s bat (29%). The six most numerous caught species, and those included as the total bat count, included: *M. myotis*, *M. daubentonii*, *M. nattereri*, *M. bechsteinii*, *B. barbastellus*, and *P. auritus,* comprising 98% of all captured animals.

Our models determined no significant effect of moonlight illumination on total bat or individual species activity, during swarming. Total bat activity, in response to changes in moonlight for netting and acoustic surveys, resulted in large *p* values (> 0.80) indicating a highly insignificant relationship (Table 1). The inclusion of site as a fixed effect indicated a significant negative correlation between the BL site and total bat activity for netting and acoustic data. Excluding *B. barbastellus*, all species activity was negatively correlated with the BL site and except for *M. nattereri* and *P. auritus* all relationships were significant. This was expected as more nets, and those of larger dimensions, were used at the FE site which has a higher abundance of bats, being the main entry point to the underground system. Activity of *B. barbastellus* exhibited a significant positive correlation with the BL site.

## Discussion

**Figure 2:**
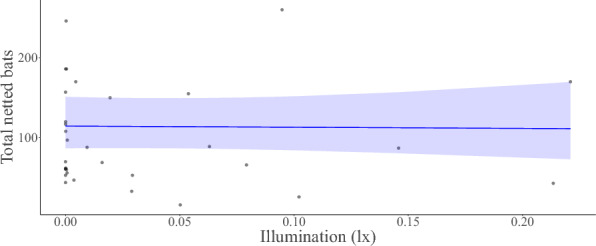
Model response estimate showing the predicted relationship between illumination and total netted bats.

**Figure 3:**
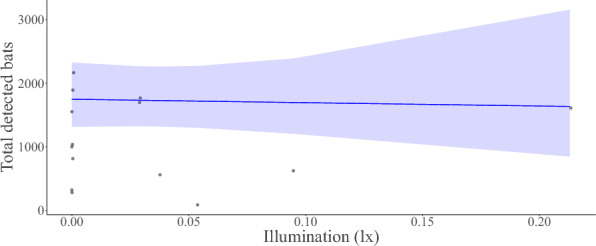
Model response estimate showing the predicted relationship between illumination and total detected bats.

**Figure 4:**
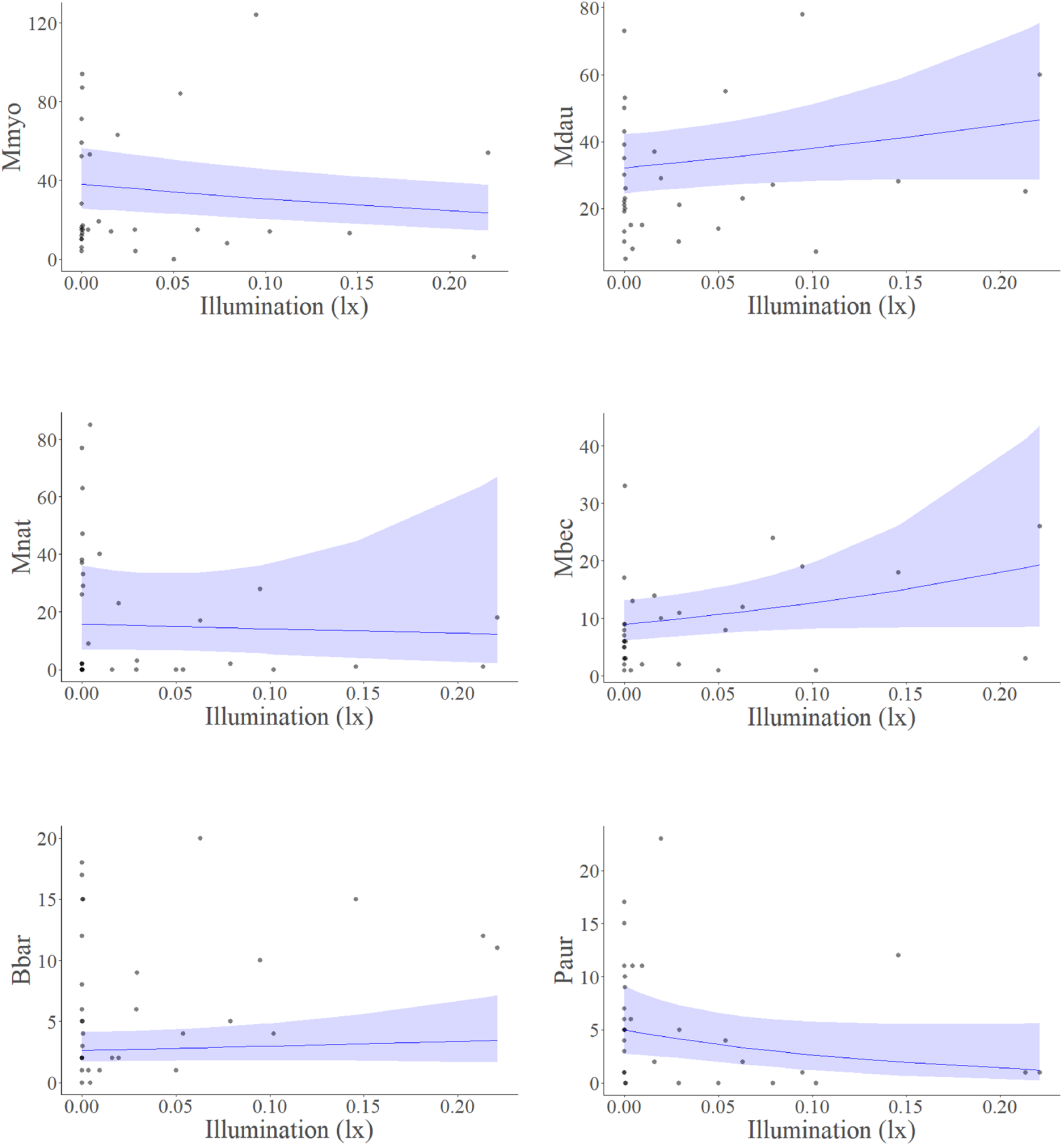
Model response estimates showing the predicted relationship between illumination and individual bat species. *Mmyo* greater mouse eared bat, *Mdau* Daubenton’s bat, *Mnat* Natterer’s bat, *Mbec* Bechstein’s bat, *Bbar* western barbastelle, *Paur* brown long eared bat.

We found no relationship between moonlight illumination and overall bat activity nor individual species activity during the autumn swarming season, thereby rejecting the theory of lunar phobia in analysed species during autumn swarming. Netted *M. myotis, M. nattereri*, *P. auritus*, and total netted and detected bats had a negative non-significant seasonal trend with moonlight; whereas the activity of the remaining three most frequently netted species netted correlated positively (Figs. 2, 4). All relationships between bats and moonlight were statistically insignificant (Figs. 2, 3, 4). Our trapping results indicate *M. nattereri* begin swarming later in the year as a result of phenology. Therefore, the observed outcome for *M. nattereri* could be a result of a peculiar data distribution rather than an actual relationship, caused by the species ecology. As such the analyses should be interpreted with caution (Fig. 4).

Our findings corroborate previous research^17,48^ evidencing a lack of lunar phobia in autumn swarming bats, but disagree with Saldaña-Vázquez and Munguía-Rosa’s extensive global meta-analysis of lunar phobia in bats, which determined a generally significant negative effect on activity^40^. Nevertheless, their meta-analysis indicates species more susceptible to lunar phobia were in the tropics where there are piscivorous, sanguivorous or frugivorous bats^20,49,50^. Such species exhibit slow flight and stationary feeding behaviours that expose them to high predation risk and consequently lunar phobia is expected. However, research has verified the phenomenon in *Myotis riparius*, a tropical insectivore, with 46.6 times greater activity on dark nights^51^. This discovery, and lack thereof in the present study, can be explained by increased relative moon brightness in the tropics. Furthermore, the meta-analysis utilised studies that assessed moon phase or moon percentage. The non-linear relationship between moon phase and illumination causes a negligible change in lux during the transition from new moon to half-moon and the majority of light increase occurs just before full moon^41^. Consequently, such studies are prone to unreliable inferences. By employing ecologically relevant levels of moonlight, and using two measurements of bat activity, our study provides robust results for the lunar phobia debate. Contemporary research assessing the influence of various abiotic factors on European bats identified moonlight caused a significant negative decrease on total bat activity and, *Pipistrellus spp., Myotis spp.,* and *Nyctalus spp.* activity^39^. Our results for *Myotis spp.* do not indicate similar findings; however, we focused on swarming whereas the authors assessing abiotic factors made no such specifications. This suggests that whilst the concept of lunar phobia in high latitude European autumn swarming vespertilionid bats included in our research may be discarded it may still be present during other stages of their phenological cycle.

The absence of lunar phobia in swarming bats may be for several reasons. Large aggregations created during swarming affords individual bats a reduction in relative predation risk through the ‘dilution effect’^15^. This enhanced safety is furthered through diminished predator success due to a difficulty in selecting an individual to predate. Swarming is one of the main periods of the year for bats to mate^52^. The importance of mating and gene flow likely outweighs the risk of predation^17^ as it is one of the strongest evolutionary drivers^53^. If moonlight can negatively influence bat activity it is highly likely to be reduced during swarming which promotes such unique behaviour. Additionally, a negative impact of moonlight on insect prey abundance which subsequently reduced foraging activity in a tropical bat species has been documented^54^. While this indirect relationship of moonlight on activity may also be present in European bats, they are likely not driven by prey presence during swarming. Further research is needed to establish the influence of moonlight levels on foraging European bats.

When designing our research we decided to abandon the study of overnight activity, limiting it to the period of higher activity^44,45^. Since our research was focused on a macro-scale phenomenon throughout the autumn season, we considered overnight behaviour (taking into account lower bat activity during the morning period) would have no impact on the obtained macro-scale results. Nevertheless, it is unlikely to use our results to interpret potential hourly variation in swarming bat activity through the night.

Our results, in agreement with other research, suggest that lunar phobia in swarming European vespertilionid bats, in high latitudes, is unlikely. By assessing six individual species (Fig. 4), we demonstrate a widespread absence of moon light avoidance behaviour. However, how this phenomenon affects bats throughout the rest of the year requires further detailed assessment. Many European species, including light phobic barbastelles, have been confirmed to emerge and begin foraging long before sunset, when lux levels are substantially higher than full moon illumination^55^. Whilst this is likely motivated by surges in prey availability at dusk^56,57^, it suggests that bats will not be discouraged by the brightest moonlight that occurs during full moon periods. Furthermore, in Germany, common noctules (*Nyctalus noctula)*, a non-swarming species, have demonstrated shifting habitat usage in response to moonlight rather than reducing activity^38^. This behaviour may occur in other European species by selecting habitats with greater canopy cover and clutter during full moon; however, research on this is lacking. Further research assessing the existence of lunar phobia in European bats outside of the autumn swarming period will provide an important clarification to a long term ecological debate.

### Supplementary Information

Supplementary Information 1.

Supplementary Information 2.

## Data Availability

The datasets generated during and/or analysed during the current study are available from the corresponding authors on reasonable request.
